# Methodology for Measurement of *in vivo* Tibiotalar Kinematics After Total Ankle Replacement Using Dual Fluoroscopy

**DOI:** 10.3389/fbioe.2020.00375

**Published:** 2020-05-05

**Authors:** Dylan J. Blair, Alexej Barg, K. Bo Foreman, Andrew E. Anderson, Amy L. Lenz

**Affiliations:** ^1^Orthopaedic Research Laboratory, Department of Orthpaedics, University of Utah, Salt Lake City, UT, United States; ^2^Department of Biomedical Engineering, University of Utah, Salt Lake City, UT, United States; ^3^Department of Physical Therapy, University of Utah, Salt Lake City, UT, United States; ^4^Scientific Computing and Imaging Institute, University of Utah, Salt Lake City, UT, United States

**Keywords:** biplane fluoroscopy, total ankle replacement, metal artifact, computed tomography, osteoarthritis, tibiotalar

## Abstract

Biomechanical data could improve our clinical understanding of failures in total ankle replacement (TAR) patients, leading to better surgical approaches and implant designs. Kinematics of the prosthetic tibiotalar joint in TAR patients have yet to be measured using dual fluoroscopy. With dual fluoroscopy, computed tomography (CT) images are acquired to track bone motion. One challenge with this approach is dealing with metal artifact in the CT images that distorts implant visualization and the surrounding bone to implant interfaces. The aim of this study was to develop a methodology to measure *in vivo* TAR kinematics using inputs of computer-aided design (CAD) models, dual fluoroscopy and CT imaging with metal artifact reduction. To develop this methodology, we created a hybrid three-dimensional (3D) model that contained both: (1) the segmented bone; and (2) the CAD models of the TAR components. We evaluated a patient following total ankle replacement to demonstrate feasibility. The patient performed a self-selected overground walk during which dual fluoroscopy images were collected at 200 Hz. *In vivo* tracking verifications were performed during overground walking using a distance calculation between the implant articular surfaces to evaluate the model-based tracking 3D solution. Tracking verification indicated realistic alignment of the hybrid models with an evenly distributed distance map pattern during the trial. Articular surface distance calculations were reported as an average of 1.3 mm gap during the entirety of overground walking. The successful implementation of our new tracking methodology with a hybrid model presents a new approach to evaluate *in vivo* TAR kinematics. Measurements of *in vivo* kinematics could improve our clinical understanding of failures in TAR patients, leading to better long-term surgical outcomes.

## Introduction

**FIGURE 1:**
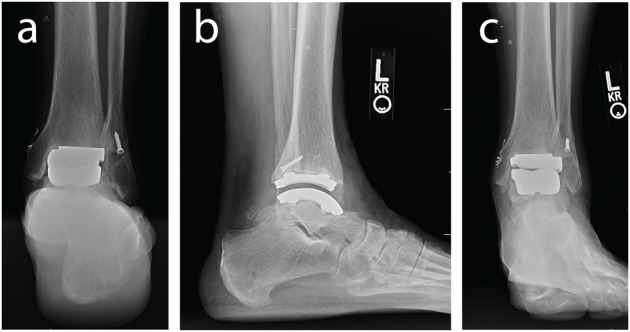
Weight bearing radiographs of the ankle and hindfoot with an implanted left TAR including: **(a)** hindfoot alignment view, **(b)** lateral view of the ankle, and **(c)** anterior-posterior view.

Ankle osteoarthritis (OA) represents a serious burden on our healthcare system. Most cases of ankle OA follow a traumatic injury, resulting in patients that are typically younger than individuals with knee OA (Saltzman et al., 2005; Brown et al., 2006; Perruccio et al., 2016). Total ankle replacement (TAR) is a surgical option to treat ankle OA (Figure 1; Saltzman, 2000; Gougoulias et al., 2010; Zaidi et al., 2013; Barg et al., 2015). Unfortunately, TAR failure rates are much higher than knee or hip arthroplasty (Labek et al., 2011). However, *in vivo* function of TAR implants is not well studied. To further understand possible modes of TAR failure, *in vivo* kinematic assessment of TAR function is needed.

TAR failure may in part be caused by altered biomechanics (Tochigi et al., 2005; Espinosa et al., 2010). Accordingly, kinematic measurements for the prosthetic tibiotalar joint could provide insight of altered movement patterns. Skin-based motion capture tracking is one of the most common ways to measure joint kinematics. Motion capture studies demonstrated reduced range of motion of the surgical TAR limb (Piriou et al., 2008; Brodsky et al., 2013; Flavin et al., 2013; Singer et al., 2013). However, motion capture lacks independent differentiation of tibiotalar and adjacent subtalar joint movement. Skin-based motion capture marker definitions typically define the calcaneus and talus as a rigid body relative to the distal tibia, due to the talus being deeply embedded beneath the skin; with measured motion being a contribution of the tibiotalar and subtalar joints within the foot (e.g., tibio-calcaneal). To accurately evaluate TAR movement independent from possible adjacent joint compensations, another experimental method involving *in vivo* medical imaging is needed.

Medical imaging techniques have been used to visualize two dimensional views of a TAR implant *in vivo*. Weightbearing and non-weightbearing lateral radiographs evaluated TAR range of motion at maximum limits of dorsi- and plantarflexion; however, this approach cannot measure dynamic tasks (Yamaguchi et al., 2011). Kinematic assessment of joint angles through dynamic experimental imaging methods currently include: (1) implantable bead tracking with imaging; and (2) single plane fluoroscopy. Implantable bead tracking is accurate to within <0.4 mm translations and <0.9 degree rotations, with beads being implanted into the bones (Bey et al., 2008). As it suggests, this is invasive and is typically only done in cadaver studies for validation (Bey et al., 2006, 2008). Single plane fluoroscopy is a model-based tracking (MBT) approach but lacks a second calibrated fluoroscopy view to accurately define 3D kinematics. Single plane fluoroscopy studies implemented videoradiography to continuously evaluate TAR dynamic range of motion and activities of daily living from a lateral view (Cenni et al., 2012, 2013; List et al., 2012). Furthermore, tracking is solely based on implant alignment and not dependent on the visible tibial or talar landmarks that could further assist in evaluating kinematic alignment on imaging methods. Utilizing single plane fluoroscopy with complex 3D geometries can be prone to increased errors in the rotational plane (Acker et al., 2011). We believe that tracking of the implant alone can likely produce worse errors for asymmetrical TAR designs, such as the Zimmer Trabecular Metal Total Ankle Replacement, therefore requiring a more rigorous experimental method.

Dual fluoroscopy (DF) and MBT quantifies kinematics through the registration of volumetric computed tomography (CT) data with dynamic images acquired *in vivo* by two fluoroscopes (Bey et al., 2008; Bedi et al., 2011). With two planes of fluoroscopy data, DF accurately measures motion of the tibiotalar and subtalar joints without skin markers (Wang et al., 2015). This technique makes no assumptions about center of rotation and does not suffer from errors due to skin motion relative to bony landmarks. Therefore, this DF method offers an accurate approach to measuring ankle kinematics of the tibiotalar and subtalar joints separately. However, to apply DF tracking methods to patients with a TAR, a CT scan is needed to create a 3D surfaces of patient specific bone geometry, ideally including the metal implant. In CT images, image artifact arises when implants are scanned, which poses technical challenges to typical DF MBT methods. Attempts to reduce CT metal artifact are available, but do not provide a suitable solution that will allow for accurate implant segmentation (Bongers et al., 2015). One experimental technique to reduce artifact is to increase tube voltage and current; however, this increases the radiation dose with only limited image quality improvement (Bolstad et al., 2018). Post-processing correction algorithms also aim to reduce the effect of beam hardening and replace it with approximated and interpolated data, which still is not as accurate as the actual implant geometry (Bolstad et al., 2018). To address metal artifact concerns, DF methods have been applied to patients following total knee replacement by using computer-aided designs (CAD) to eliminate the need for CT scans; yet, knee kinematics were solely based on implant geometries (Hanson et al., 2006). Modern TAR implants have a lower profile with planar symmetries, making it difficult to have kinematic confidence based on CAD implant tracking alone. There is still a need to develop post-processing methodologies for MBT methods to support DF evaluation of *in vivo* foot and ankle motion in patients with TAR.

The aim of this study was to develop a methodology to measure *in vivo* TAR kinematics for patients with a Zimmer Trabecular Metal Total Ankle Replacement using inputs of CAD models, DF and CT images. The fixed bearing design of this TAR implant has not been evaluated previously and warrants *in vivo* experimental investigation, but first a methodology must be established. To develop this methodology, we created a hybrid 3D model that contained both: (1) the segmented bone; and (2) the CAD models of the TAR components. This hybrid model was then used for tracking via MBT as a single rigid body per bone. Our objective was to develop and implement the MBT protocol in a single patient to demonstrate the feasibility of this methodology by evaluating *in vivo* distance calculations between the implant articular surfaces to evaluate the model-based tracking 3D solution. We hypothesize that an average articular surface distance between the tibial implant fixed bearing polymer surface and talar implant would range from 0.5 mm surface penetration to a 1.5 mm gap, will indicate realistic implant alignment within the capabilities of *in vivo* DF imaging system. This method will ultimately lead to studies including larger cohorts of patients with TAR implants that should improve understanding of the biomechanical kinematic function for this procedure.

## Materials and Methods

### Participant

A single case study was collected for a human participant (male, 77 years of age, 23.1 kg/m^2^ BMI) who had received a Zimmer Trabecular Metal Total Ankle Replacement 1.5 years prior. The participant provided full written and verbal consent for this IRB approved study (University of Utah #65620) that adhered to the Helsinki Declaration. The participant was screened using clinical foot and ankle radiographs to ensure his prosthesis components were stable and appropriately aligned with no clinical signs of implant loosening (Figure 1). At the time of testing, the participant was unremarkable for signs of TAR failure with no patient reported pain.

### Data Acquisition

#### Dual Fluoroscopy Motion Capture

**FIGURE 2:**
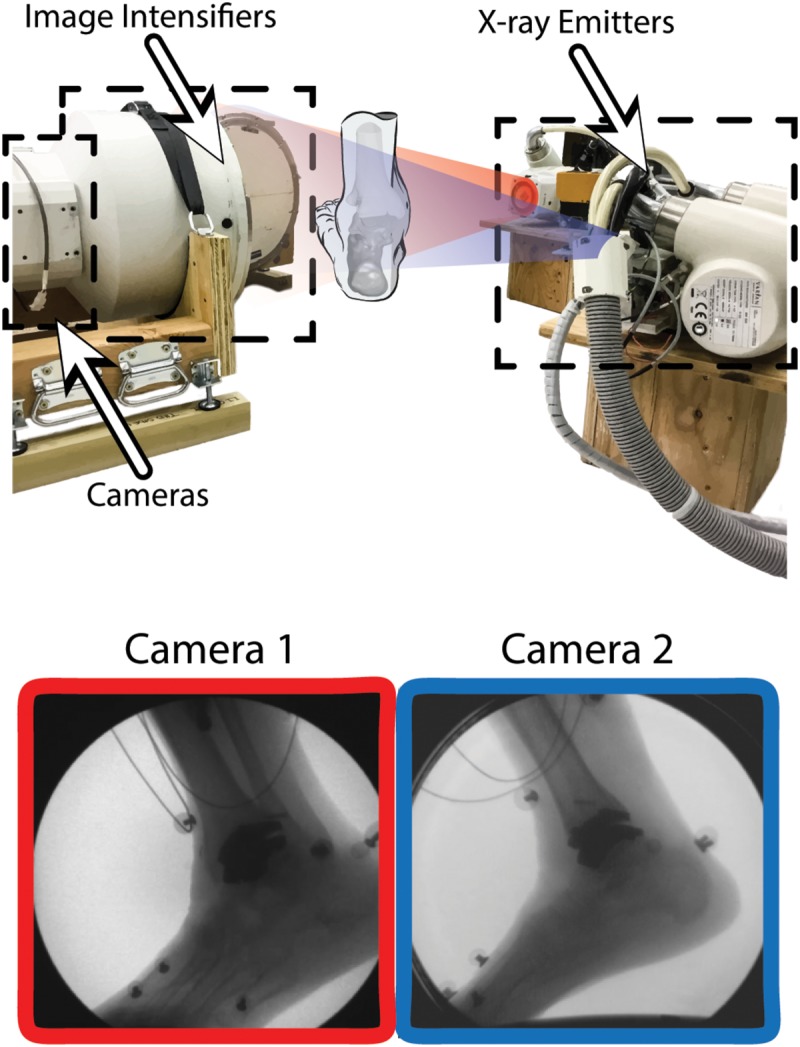
Dual fluoroscopy (DF) system setup for overground walking with two X-ray emitters and two image intensifiers with high-speed cameras oriented ≈90 degrees from one another. Two synchronized DF views (Camera 1 and 2) are shown during overground walking toward the toe-off phase of gait. Wires from electromyography sensors can be seen in the DF image.

A custom dual fluoroscopy (DF) imaging system was implemented to obtain two calibrated views acquiring videoradiography for frame-by-frame imaging (200 Hz). The DF system included two X-ray emitters, two image intensifiers, and high-speed cameras, placed approximately orthogonal from one-another with 608 × 600 resolution (Radiological Imaging Services, Hamburg, PA, United States) (Figure 2). The DF system energy settings were 68 kVp and 1.8 mAs. This system was previously validated to submillimeter accuracy for translation and rotation in a cadaver study (Wang et al., 2015) and used for *in vivo* ankle studies (Roach et al., 2016, 2017). A static trial was collected with the participant standing within the DF field of view. Next, an overground walking trial was performed at the participant’s self-selected walking speed. The DF system was positioned and synced to acquire in-ground force platform data to identify gait events during the walking trial (AMTI OR6 series; Watertown, MA, United States). The DF system was temporally synced with two force platforms using an external trigger (Wang et al., 2015).

#### Computed Tomography

A supine computed tomography (CT) scan was acquired (SOMATOM Definition AS: Siemens Medical Solutions, Malvern, PA, United States) with a field of view (512 × 512 acquisition matrix) that encompassed the distal foot to the proximal tibia at a 0.6 mm image slice thickness with isotropic voxel size. Tube voltage and current were 90 kVp and 45 mAs respectively using CareDose^TM^. The CT scan exposed the participant to ionizing radiation. The effective dose equivalent for this lower extremity CT scan was 0.9 mSv, which is ≈29% of the background radiation an average person in the United States receives annually from naturally occurring sources (≈3.1 mSv per year). An iterative metal artifact reduction algorithm was also applied (Siemens iMAR^®^).

### Initial Segmentation and Digitally Reconstructed Radiographs

**FIGURE 3:**
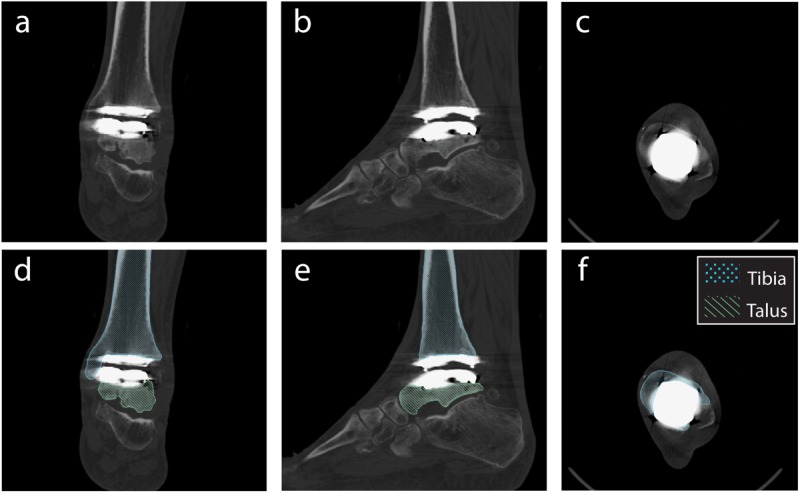
Planar CT image slices using the metal artifact reduction algorithm (Siemens iMAR^®^). Planar views demonstrated in: **(a,d)** Coronal, **(b,e)** Sagittal, and **(c,f)** Transverse planes. Segmentation shown for the tibia and talus **(d–f)**.

From the obtained CT scan, the tibia and talus were segmented by outlining the boundary of the cortical bone in a commercially available segmentation software (Amira 6.2: Visage Imaging). Tibia and talus smoothed 3D surfaces were generated in the segmentation software. iMAR has been shown to be more effective than other image processing and filter algorithms for minimizing metal artifact in CT imaging of Zimmer Trabecular Metal Total Ankle implants (Khodarahmi et al., 2018). Still, difficulties arose with segmentation of cortical bone that was adjacent to the implant; notably, iMAR did not eliminate metal artifact completely (Figure 3). Implant-induced artifacts in high-density metal regions reported here were consistent with the literature, which noted the inability to perfectly correct for beam hardening (Khodarahmi et al., 2018). To circumvent this issue, CAD implant models, obtained from the manufacturer, were imported into the segmentation software.

**FIGURE 4:**
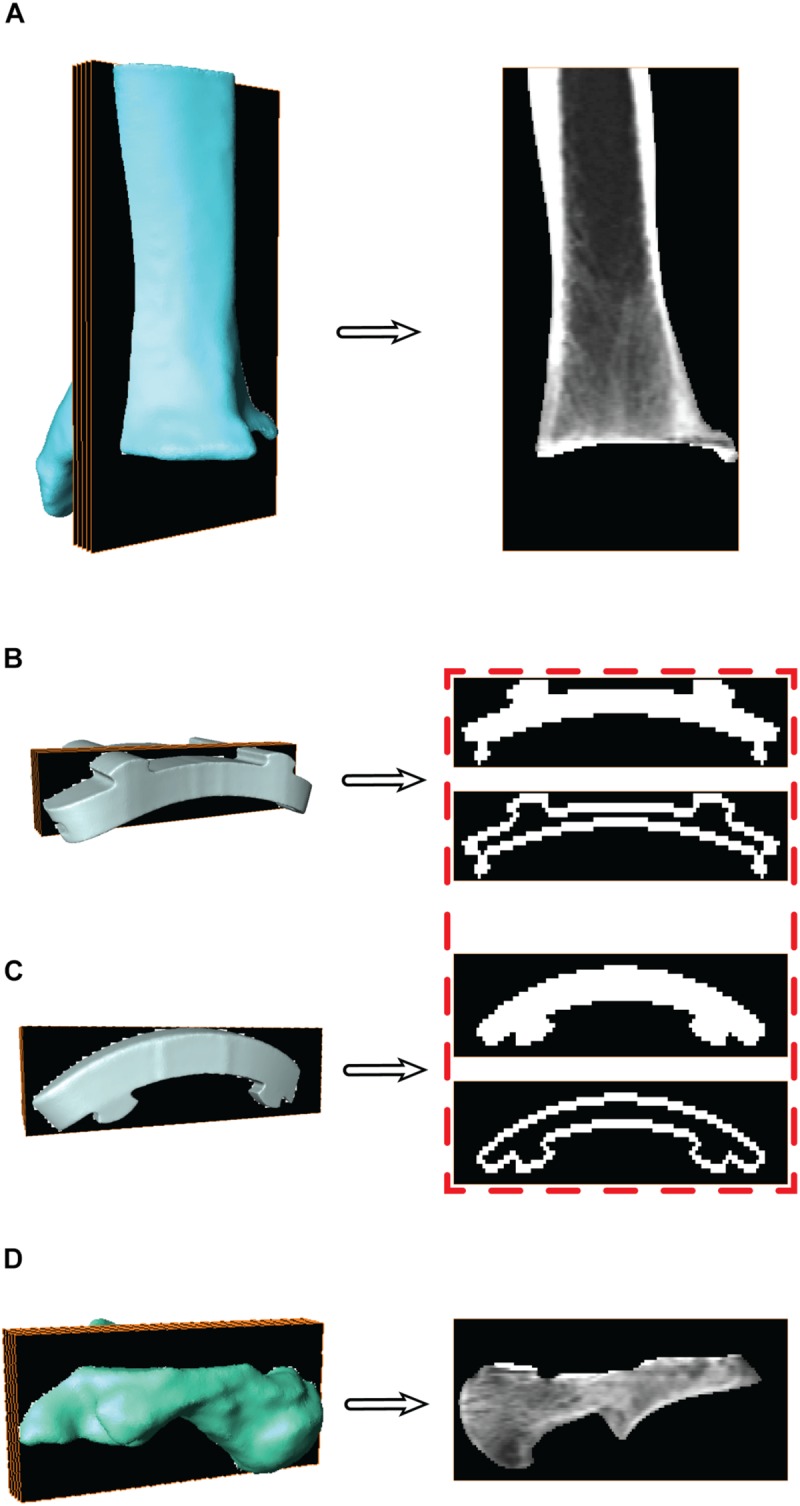
Surface models with a representative sagittal slice show the isolated digitally reconstructed radiographs (DRRs) for the **(A)** tibia, **(B)** tibial component, **(C)** talar component, and **(D)** talus. Red box highlights the additional step to hollow the implants for improved edge contrast to control for oversaturation during model based tracking (MBT).

For the individual bones, digitally reconstructed radiographs (DRRs) were created using the segmentation software and are the images used for MBT. DRRs were semi-automatically co-registered with the DF images using previously-published MBT software (Bey et al., 2006, 2008). With the CT image stack and the bone surface models, the bone was isolated from the CT images (Figures 4A,D). This was done by converting the 3D surfaces to slice-by-slice binary image stacks: pixel values were assigned a value of 1 (white) where bone was located and 0 (black) everywhere else using a binary image tool in the segmentation software. Then, with the converted 3D surface to binary image stack and the original CT image stack, an arithmetic operation was performed to successfully isolate the bone within the CT image stack.

For the CAD implants, the same process used to generate DRRs was applied to track the implant components similarly to the bones via MBT. The CAD models were converted to slice-by-slice binary image stacks. The binary images were altered to mimic the dense boundary of cortical bone: by hollowing out the binary implant images (Figures 4B,C). Hollowing the implants defined the edges, which was deemed necessary to aid in tracking since MBT employs an edge detection auto-tracking algorithm (Bey et al., 2006, 2008). The hollowing of these binary implant images was performed in a systematic manner via the segmentation software where all the slices were selected, and a threshold selected to isolate the implant. Once only the implant was selected, then the internal regions were deselected. This effectively hollowed out each binary image within the implant image stack by isolating only the edges of the implant.

### Static Tracking of Individual Bones and Implants

**FIGURE 5:**
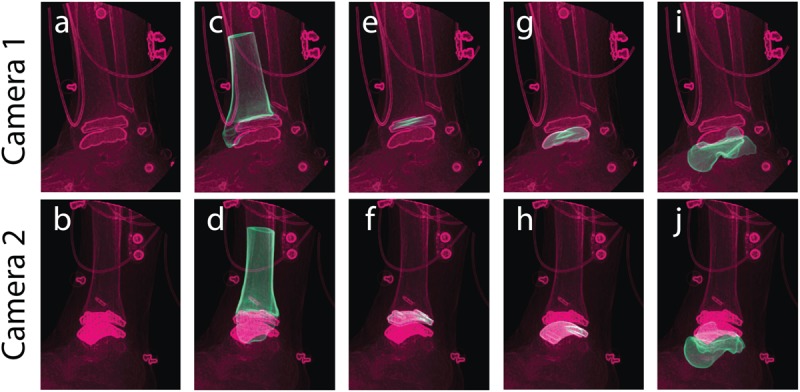
Tracking was performed in a static standing position for bone and implant models to yield individual structure positional and rotational solutions to establish a combined hybrid model. Camera 1 and camera 2 show the two calibrated DF views for a single frame in the static trial. Multiple views of the same static frame demonstrate the raw DF images **(a,b)**, and individual tracking solutions for the: tibia **(c,d)**, tibial component **(e,f)**, talar component **(g,h)**, and talus **(i,j)**.

With the DF static standing trial, DRRs for the tibia and talus were tracked via MBT, as well as the mimicked DRRs for their respective implants. Each object (tibia, tibial component, talar component, and talus) was tracked for up to 20 frames of the static trial (Figure 5). With these tracked frames, positional and rotational information was extracted to establish locations of the implants with respect to the bones. The positions and orientations of the tibia, talus, and their respective implants were averaged across all tracked frames to use as a mean static definition. The mean static definition established a coordinate relationship between the implant and its respective bone. These static positions were then used in the next step of creating a hybrid model, linking a bone with its respective implant as a rigid body.

### Hybrid Model Development

#### Hybrid Model Transformations

To establish a hybrid model containing bone and implant components, the first step was to use the mean location in the static position of the individual bone and implant positions and orientations. Coordinate systems were defined for the bones and implants in three coordinate system domains: CT derived surface, MBT dual fluoroscopy, and CAD model implant reference frame. The extracted MBT positions and orientations identified the bone/implant in their arbitrary MBT vector space. A position and orientation of the bone/implant in their arbitrary model vector space or CT derived surface was also established. These coordinate systems’ origins were defined by the bounding box corners (obtained from the segmentation software during DRR generation). By utilizing a global coordinate system (a 3-dimension basis formed by {[100]^*T*^,[010]^*T*^,[001]^*T*^}) rotation and translation between the CAD model coordinate systems and the MBT coordinate systems were then achieved. Transformations were obtained from the CT derived surface coordinate systems to the global coordinate system via a composition of translations and rotations, respectively, where *t* indicates the translation vector, *R* indicates the rotation matrix, and *T* indicates the transformation matrix:

$$t_{C\,T\to g\,l\,o\,b\,a\,l}=O_{g\,l\,o\,b\,a\,l}-O_{C\,T};$$

RC⁢T→g⁢l⁢o⁢b⁢a⁢l=[xC⁢Tg⁢l⁢o⁢b⁢a⁢l⁢yC⁢Tg⁢l⁢o⁢b⁢a⁢l⁢zC⁢Tg⁢l⁢o⁢b⁢a⁢l]=I;

(1) $$\Rightarrow T_{CT\to global}=\left[\begin{array}{cc} R_{CT\to \text{global}} & t_{CT\to global}\\ 0 & 1 \end{array}\;\right];$$

where *O* indicates the origins, and {*x, y, z*} indicate the 3-dimensional coordinate vectors. The rotations were obtained by projecting the global coordinate systems onto the CT coordinate systems which simplified to the identity matrix, *I*, since the bounding boxes from the segmentation software were composed of an identical 3-dimensional basis as described above for the global coordinate system. Similarly, the transformations from the global coordinate system to the MBT derived coordinate systems were obtained via a composition of translations and rotations, respectively:

$$t_{g\,l\,o\,b\,a\,l\to M\,B\,T}=O_{M\,B\,T}-O_{g\,l\,o\,b\,a\,l};$$

Rg⁢l⁢o⁢b⁢a⁢l→M⁢B⁢T=[xg⁢l⁢o⁢b⁢a⁢lM⁢B⁢T⁢yg⁢l⁢o⁢b⁢a⁢lM⁢B⁢T⁢zg⁢l⁢o⁢b⁢a⁢lM⁢B⁢T]

(2) $$\Rightarrow T_{global\to MBT}=\left[\begin{array}{cc} R_{global\to MBT} & t_{global\to MBT}\\ 0 & 1 \end{array}\right].$$

The main differences arise with: (1) the order of origins when calculating the translation vector; and (2) instead of simplifying to the identity matrix, the rotation matrix simplified to a composition of row vectors (the MBT basis vectors) after projecting these onto the global coordinate system. Ultimately, composing these transformations (from Equations 1 and 2) resulted in the desired finalized transformation to transform MBT derived surfaces into the CT space:

(3) $$T_{C\,T\to M\,B\,T}=T_{g\,l\,o\,b\,a\,l\to M\,B\,T}\cdot T_{C\,T\to g\,l\,o\,b\,a\,l}.$$

By knowing these transformations, the bone models were transformed to the MBT vector space. After both the bone and respective implant were in MBT space, they were then transformed back to the respective bone’s (using the result from Equation 3) model space via:

(4) $$T_{M\,B\,T\to C\,T}={\left(T_{C\,T\to M\,B\,T}\right)}^{-1}.$$

**FIGURE 6:**
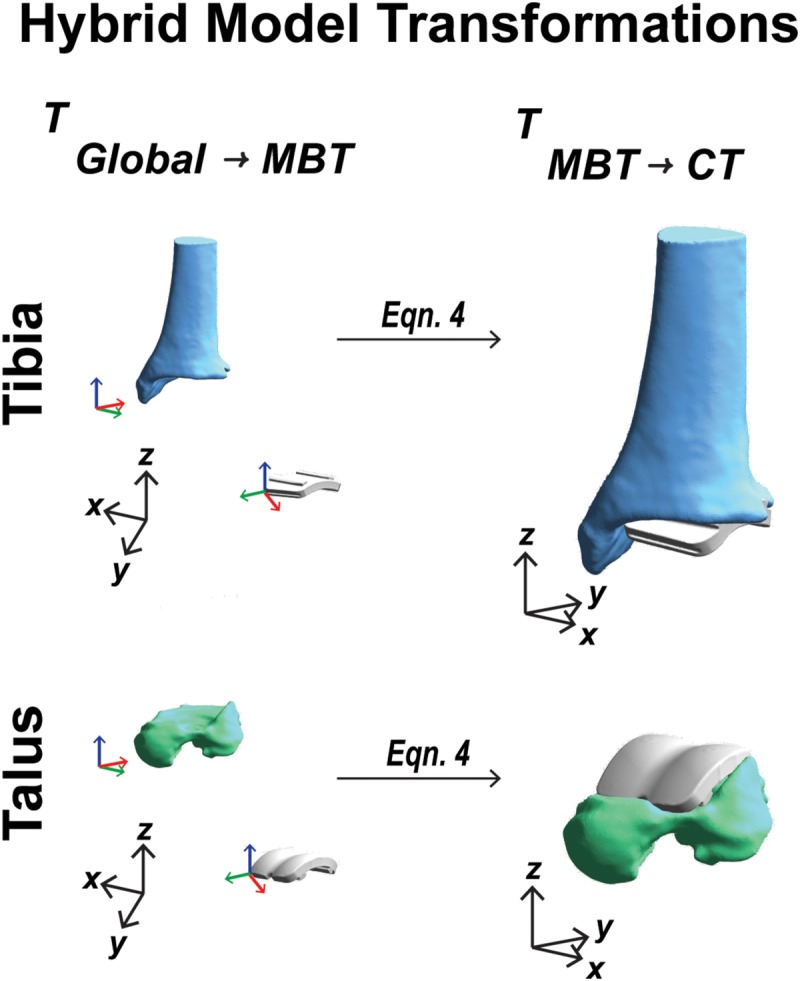
Visual representation of the coordinate system transformations performed from global vector space, represented by black axes, through model-based tracking space to finalized CT vector space for a tibia and talar hybrid model transformation.

These were transformed back to the bone’s model space to align with the CT images. The bones and implants now resided in the CT coordinate system and sequential steps for creating a hybrid DRR for dynamic tracking could follow. The complete sequence of transformations started in the global coordinates and ended in the CT coordinate system (Figure 6).

### Re-segmentation at Bone/Implant Interface

**FIGURE 7:**
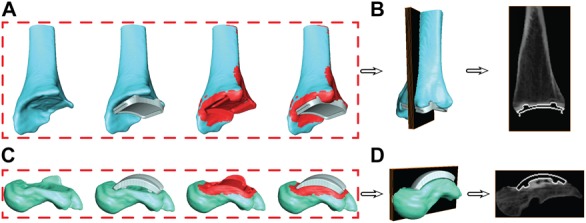
Progression of the segmentation process shown for the tibia **(A)** and talus **(C)** hybrid models. Initial segmentation with the aligned implant shows gaps between the bone and implant. Re-segmentation shown in red was performed to create a solid bone/implant interface. Finalized hybrid surface models were merged with the CT data to create a complete model of the bone and hollowed implant for the tibia **(B)** and talus **(D)** to develop digitally reconstructed radiographs for hybrid model dynamic tracking.

With the known location of the implant with respect to the bone, the bone segmentation was re-evaluated. Regions with metal artifact on the CT scan were re-segmented to interpolate the empty space between the original bone boundary and the implant to create a solid model. This process was completed in the segmentation software by adding bone material up to the boundary of the implant on both the tibia and talus. Following re-segmentation, the model represented a surface with a full contact bone-to-implant interface (Figures 7A,C).

### Hybrid Digitally Reconstructed Radiograph Models

During the creation of the hybrid DRR models, the pixel intensities of the images were adjusted separately for both the bone and implant. The intention of this additional step was to create a balanced visualization of bone vs. implant brightness. Bone intensities were decreased by 0.6 (60%) to reduce intensity compared to their respective implants. Similarly, implant intensities were magnified by 100. Finalized hybrid model DRRs were then ready for dynamic MBT (Figures 7B,D).

### Dynamic Tracking Evaluation

#### Model-Based Tracking

With the developed hybrid model, previously validated methods for MBT were implemented to track the tibia/implant and talus/implant models during an overground walking trial (Bey et al., 2006; Wang et al., 2015; Roach et al., 2016). Semi-automated tools within the tracking software were utilized such as edge detection and Sobel image processing to align DRRs in the two calibrated DF views in each frame of the trial (MtwTesla). Three-dimensional surface dynamic visualization was implemented to evaluate initial tracking results (PostView 2.0; University of Utah; Salt Lake City, UT, United States). Hybrid models were imported into PostView and the kinematics tool was used to drive models with MBT results. 3D visualization provided feedback to the individual performing semi-automated tracking on the appropriateness of bone positions. For example, in the 3D model, out of plane erroneous rotations and translations could be visually seen and corrected for to yield a physiologically feasible solution.

### Tracking Method Verification

Previous studies implementing DF kinematic analysis were validated with a cadaver model and implanted beads to compare MBT with bead tracking methodologies to quantify error and report the accuracy of our DF system (Wang et al., 2015). While the DF system used in this study has not changed in validation, we created an additional *in vivo* tracking verification customized to account for image processing in the setting of DF of patients with TAR.

#### Articular Surface Distance Analysis

A common method for tracking verification in our experimental post-processing protocols is to determine if two or more bones are overlapping with one another within joints of 3D surface models driven by tracking results. If bone-to-bone motion yields bone overlap within the articular surfaces, the MBT solution will be re-examined to ensure proper DRR alignment until all bone overlap has been eliminated. However, in the TAR population, bony articular surfaces have been replaced by the TAR prosthesis. The Zimmer Trabecular Metal TAR includes a polymer insert that rigidly attaches onto the tibial component of the implant as a fixed bearing design. The CAD model for the polymer insert was imported and rigidly attached to the tibial/implant hybrid model. Using the surface distance tool in PostView, faces of the polymer articular surface and talar articular surface were selected to calculate articulation distances throughout the overground walking trial. An average of all distances within the articular surface was evaluated. An average acceptable range of <0.5 mm surface penetration to <1.5 mm distance of a gap was defined as a reasonable tracking solution. Small deformation of the polymer insert provided rationale to allow minimal surface penetration. Distance maps were also evaluated qualitatively for an even distribution of distance throughout the articular surface.

### Kinematic Analysis

#### Bone/Implant Coordinate System Definitions

To calculate joint angle kinematics, coordinate systems were first defined for the: (1) tibia hybrid model and (2) talus hybrid model.

**FIGURE 8:**
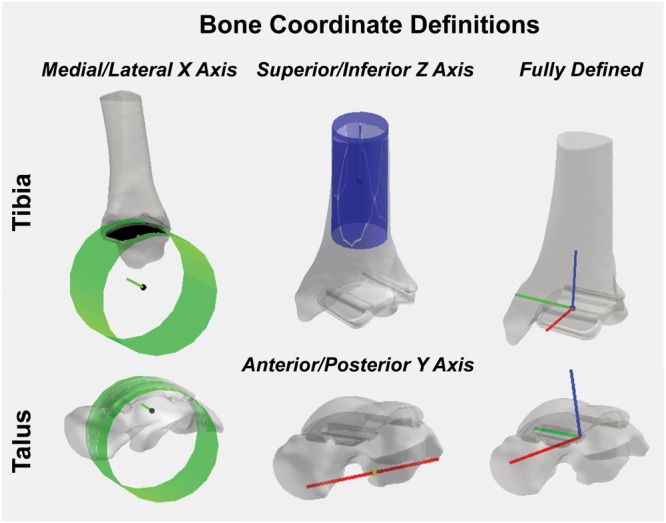
Tibial and talar hybrid model coordinate system definitions based on an anatomical and implant landmark approach. Cylindrical fit approaches were used to identify medial/lateral axes based on the implant articular surface curvature. A tibial shaft cylindrical fit defined the superior/inferior axis. Anatomical landmarks on the inferior surface of the talus defined the anterior/posterior axis. Cross-products yielded fully defined hybrid model coordinate systems.

For the tibia hybrid model, the landmarks were selected: (1) the tibial shaft; (2) the distal articulating surface of the tibial component; and (3) the distal-medial implant edge. A cylinder was then fit to the distal articulating surface (Figure 8). With this cylindrical fit, a vector was aligned through the center of the cylinder to define a medial-lateral axis for our coordinate system. The base of this medial-lateral axis was defined as the temporary origin. Another cylinder was fit to the tibial shaft. With this cylindrical fit, a vector was similarly aligned through the center of the cylinder to define a temporary superior-inferior axis for our coordinate system. The origin was defined as the intersection of the medial-lateral axis translated superiorly along the superior-inferior axis until it fell on the plane defined by the base of the tibial implant. The anterior-posterior axis was obtained by taking the cross-product of these two vectors, where *v* indicates the vectors in each axes direction:

(5) $$v_{S\,I,t\,e\,m\,p}\times v_{M\,L}=v_{A\,P}.$$

With two fully defined bases vectors to define our coordinate system, a final cross-product between the medial-lateral and anterior-posterior coordinate vectors finalized our superior-inferior coordinate vector, effectively creating three mutually orthogonal base vectors to completely establish a left-handed coordinate system for our hybrid tibia surface:

(6) $$v_{M\,L}\times v_{A\,P}=v_{S\,I}.$$

A left-hand coordinate system was chosen because the patient had a TAR on the left limb. For patients with a right limb TAR, a right-hand coordinate system would be used.

For the talus, the landmarks selected were: (1) the proximal talar component curvature; (2) an anterior spline; and (3) a posterior spline. A cylinder was fit to the talar component curvature (Figure 8). With this cylindrical fit, a vector was aligned at a quarter of the cylinder radius to define a medial-lateral axis; the base of this vector was defined as our origin. Then, by creating a vector between the two spline selections, a temporary anterior-posterior axis was defined by translating its base to the origin of our coordinate system. Similarly, as was done for the tibia, by taking the cross-product of the two, we finalized an orthogonal superior-inferior axis. Lastly, with two fully defined base vectors to define our coordinate system, a final cross-product between the superior-inferior and medial-lateral coordinate vectors finalized our anterior-posterior coordinate vector, effectively creating three mutually orthogonal base vectors to completely establish a left-handed coordinate system for our talus hybrid model.

### Joint Angle Kinematics

Dynamic joint angles were calculated as previously described using the Grood and Suntay method to assign: plantar/dorsiflexion, internal/external rotation and inversion/eversion between the tibia and talus (Grood and Suntay, 1983; Wang et al., 2015). Dynamic joint angles were filtered with a fourth-order bidirectional low-pass Butterworth filter using a residual analysis method to select a cut-off frequency of 10 Hz. Kinematics were normalized to percent stance by identified gait events from heel-strike to toe-off for overground walking based on 5% force detection of maximum from the in-ground force platform. Our TAR kinematic results for one patient were compared to confidence intervals of healthy controls previously collected using the identical DF system (Roach et al., 2016).

## Results

### Dynamic Tracking Verification

**FIGURE 9:**
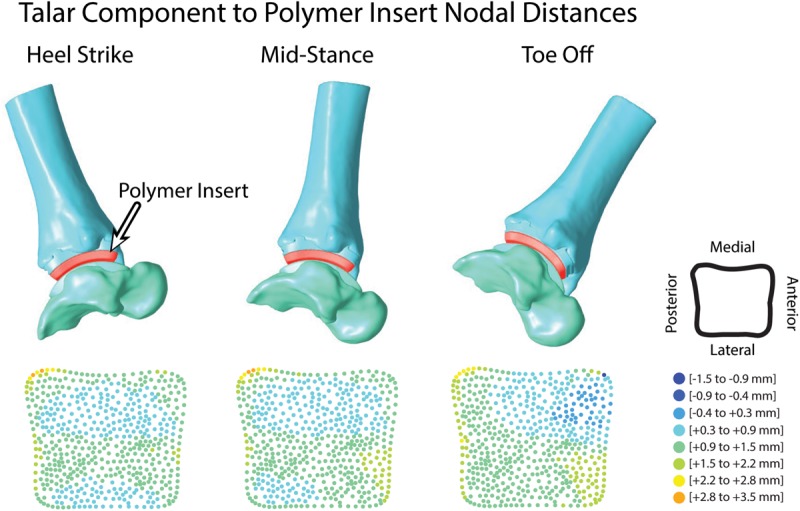
Hybrid models from hybrid model tracking solutions during walking with polymer insert shown in red. 3D model positions from MBT solutions shown at heel strike, mid-stance and toe off with corresponding distance maps of the articular surface relationship between the polymer insert and talar implant.

Articular surface distance calculations were reported as an average of 1.3 mm during the entirety of overground walking. Surface distances were evaluated for each tracked frame to assess evenly distributed distance map patterns during the trial (Figure 9). At heel strike, surface distances showed two anterior-posterior regions of minimal contact (−0.4 to 0.3 mm) along the bicondylar ridges of the talar implant and continued through mid-stance. At toe off, increased contact was seen in the anterior-medial articular region.

### Kinematic Analysis

**FIGURE 10:**
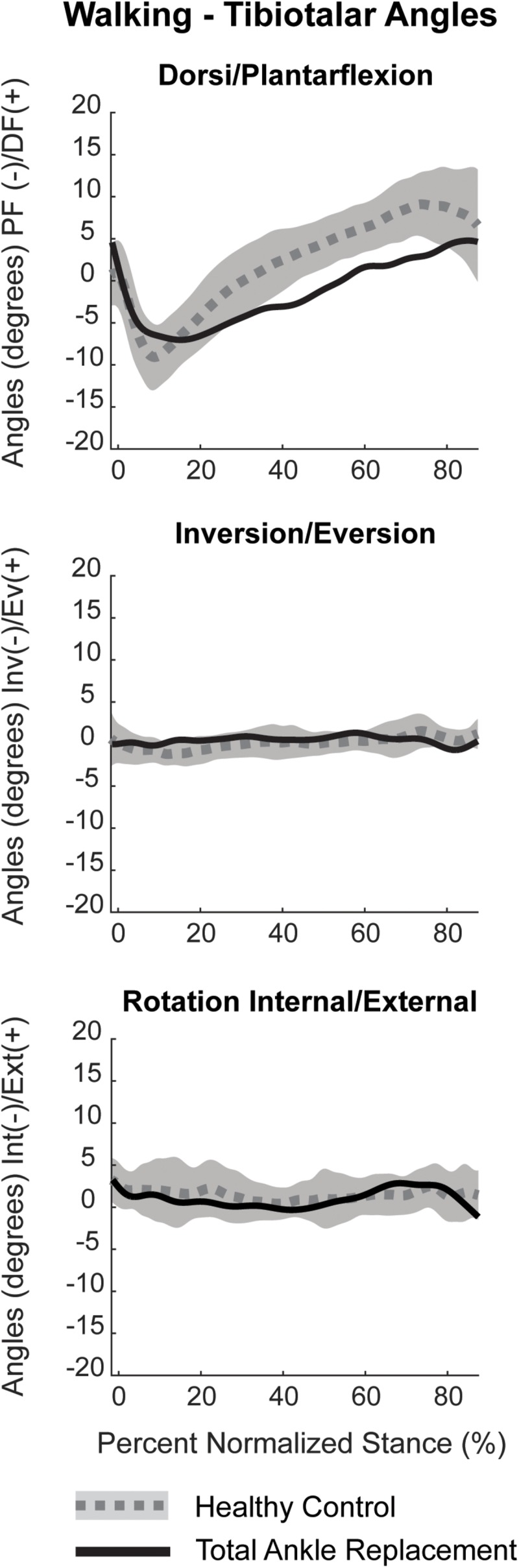
Planar tibiotalar kinematics (dorsi/plantarflexion, inversion/eversion, and internal/external rotation) for the TAR participant compared with confidence intervals of healthy controls analyzed using DF during overground walking. Walking kinematics are normalized to percent stance from heel strike to toe off.

Dorsiflexion/plantarflexion tibiotalar kinematics for the TAR patient trended toward that of healthy controls. However, the TAR patient exhibited reduced peak plantarflexion in early stance and minimal dorsiflexion in late stance (Figure 10). During mid-stance, the angular rate of change from plantarflexion to dorsiflexion was reduced compared to healthy controls. Inversion/eversion and internal/external rotation kinematics for the TAR patient fell within the confidence intervals for healthy controls.

## Discussion

To date, no literature reports the use of DF to evaluate *in vivo* kinematics in patients with a TAR. When using imaging techniques to study *in vivo* kinematics of a joint replacement, metal artifact on CT images makes current methods of anatomy reconstruction challenging. The aim of this study was to develop a methodology to measure *in vivo* TAR kinematics using inputs of CAD models, DF and CT images. To develop this methodology, we created a hybrid 3D model that contained both: (1) the segmented bone; and (2) the CAD models of the TAR components. Hybrid models were used to perform MBT and calculate TAR kinematics with the reported process that was modified from a previously validated methodology (Wang et al., 2015).

Metal artifact on CT imaging modalities can pose technical and clinical challenges in the evaluation of the appearance of a tissue that is adjacent to an implant. Metal artifact reduction algorithms have been developed to reduce the effect of beam hardening and photon starvation caused by metal (Aissa et al., 2017; Bolstad et al., 2018). The type of metal used in the implant affects metal artifact; for example, cobalt chromium alloys demonstrate greater artifact than titanium alloys (Bolstad et al., 2018). With our developed method, Siemens iMAR^®^ was applied to the CT scan of the patient with a TAR. Qualitative improvements were observed between the raw CT scan and after iMAR application; yet, distortion surrounding the implants was still present. Metal artifact prohibited direct segmentation of the implants. Thus, we developed a process that leveraged the CAD models to provide information concerning the shape of the implants. The tibia and talus were segmented up to the region where artifact was present (Figure 3). However, this left the bones with unusual boundaries near the distal tibia and proximal talus. Initially, hybrid models were created using the static MBT method for alignment and developed hybrid DRRs were used for dynamic tracking. Dynamic tracking proved difficult due to the lack of a solid bone-implant interface which created additional false edges on the DRRs and worsened the successful application of the tracking edge detection algorithms. Our solution was to re-segment the bone-implant interface (Figure 7). Resulting hybrid models with the improved bone-implant interface resolved MBT issues. Throughout the development of hybrid models, it was determined that while iMAR did not completely resolve artifact on the CT images, it did improve the ability to segment cortical bone in the surrounding region.

Although the DF system used herein had been previously validated (Wang et al., 2015), modifications made to the previous methodology motivated the necessity of an additional verification process to assess TAR tracking results. Metal artifact is not a concern when using DF because image intensifiers are commonly shielded with mu-metal to minimize distortion (Nickoloff, 2011), however, our typical method for assessing bone-to-bone overlap to evaluate the quality of the MBT was modified due to the implanted TAR prostheses (Wang et al., 2015). Hybrid models driven with tracking results yielded a large gap between the implants where a polymer insert articulates in this particular Zimmer design (Martinelli et al., 2017). The polymer insert is rigidly attached to the tibial TAR component in a fixed bearing style design. While this polymer insert is not visible on DF images, we could import the polymer into our 3D visualization method that was driven with MBT solutions. Overall, the addition of the polymer insert improved the quality of the tracking for each hybrid model by quantifying articular distances and evaluating the final solution before calculating kinematic results.

We developed the methodology in preparation for longitudinal studies to examine the biomechanics of TAR *in vivo*. It is possible that implant wear could affect calculations of implant kinematics since an implant that has undergone wear would have different geometry than the original CAD model. The Zimmer Trabecular Metal Total Ankle design consists of a Ti-6 Al-4V tibial component, a fixed-bearing electron beam crosslinked GUR 1050 ultra-high molecular weight polyethylene (UHMWPE) articular surface, and a highly polished CoCrMo alloy talar component. Excessive wear to the UHMWPE fixed bearing is of greatest concern. A previous study performed *in vitro* gravimetric wear testing of this specific implant design to closely mimic *in vivo* kinematic and kinetic conditions (Kincaid et al., 2013). The load and motion waveforms were applied at 1.1 Hz for a total test duration of 5 million cycles (Mc). The mean volumetric wear rate with 95% confidence limits was 3.3 ± 0.4 mm^3^/Mc (Kincaid et al., 2013). To convert this reported volumetric wear rate to an *in vivo* scenario overtime, average step activity of patients receiving a TAR must be considered. After surgical treatment of ankle OA with a TAR, patients increased their activity with an average of 4,619 steps per day at a 36 month post-surgical follow-up (Shofer et al., 2019). To overestimate daily steps, we will consider a TAR patient is averaging 5,000 steps per day which equates to 1.825 Mc/year. Given the reported mean volumetric wear rate, that yields an annual rate of wear equaling 6.023 mm^3^/year. Provided the CAD models, a contact surface area for the UHMWPE can be calculated on a patient specific basis with the known implant size. For our patient, the articular surface area was 945 mm^2^. Our patient was studied 1.5 years post-operatively, therefore the articular surface of his implant was estimated to show an average linear contact wear of 0.0096 mm. If the same patient was studied 10 years post-operatively, the derived linear contact wear would be an average of 0.0637 mm. Our DF system mean translational and rotational precision has been previously reported to be 0.3 ± 0.12 mm and 0.63 ± 0.28 degrees, respectively (Wang et al., 2015). Therefore, any wear of the implant would not be within the imaging capabilities and would not impact the ability to utilize this developed methodology for tracking *in vivo* kinematics. This conclusion is further supported by literature investigating total knee arthroplasty (TKA) wear with x-ray evaluation. TKA implants with an electron beam crosslinked UHMWPE bearing were found to have no detectable wear on x-rays 15 years after implantation (Jenny and Saragaglia, 2019). Studies performing *in vitro* wear tests have been similarly conducted for TKA implants and report a mean volumetric wear rate of 4.0 ± 1.0 mm^3^/Mc (Brockett et al., 2018). The data in TKA implants further supports the conclusion that implant wear is minimal overtime and will be undetected when using x-ray or fluoroscopy based imaging techniques.

Reportedly, only single plane fluoroscopy has been used to evaluate motion in patients with a TAR (Cenni et al., 2012, 2013; List et al., 2012). In these studies, the TAR implant design studied had less symmetries and more prominent landmarks to use for MBT alignment than the TAR studied in this developed methodology. Therefore, the lack of DF MBT methods in patients with a Zimmer Trabecular Metal Total Ankle Replacement provided motivation to develop a robust method to evaluate *in vivo* kinematics in this highly symmetrical implant design. Our method presents an experimental and post-processing workflow to report TAR kinematics with a high level of confidence in the 3D joint angle outputs.

Some limitations related to the developed methodology should be highlighted. First, the validation study to assess the DF and MBT accuracy was performed in a cadaver model with intact tibiotalar and subtalar anatomy (Wang et al., 2015). A validation study could be performed in a cadaver model with the specific TAR model to further investigate the method’s accuracy. Secondly, this is currently a single case study for implementing the new methodology. Clinical interpretation of the preliminary kinematic results is not a focus of this study, even though some dorsi/plantarflexion variations from healthy controls were noted. Future studies will recruit additional patients and implement this methodology to draw clinical conclusions in a larger set of patients with TAR.

## Conclusion

We reported a methodology of using CAD models, CT imaging with metal artifact reduction, and MBT to create a bone and implant hybrid model for tracking *in vivo* kinematics. We evaluated a single patient with a unilateral TAR to demonstrate the feasibility of this approach. Tracking verification indicated reasonable alignment of the hybrid models, thus demonstrating the feasibility of the described approach for tracking TAR implant motion using DF. Measurements of *in vivo* kinematics in patients with TAR could improve clinical understanding of failures, leading to better surgical approaches and implant designs.

## Data Availability Statement

The datasets generated for this study are available on request to the corresponding author.

## Ethics Statement

Full written and verbal consent was obtained from the participant for this IRB approved study (University of Utah #65620) that adhered to the Helsinki Declaration and permitted the publication of any potentially identifiable images or data included in this manuscript.

## Author Contributions

AL, DB, and AA conceived and designed the experiments. AB screened potential participants. AL, DB, and KF acquired the experimental data. AL and DB carried out the analysis, interpreted the data, and wrote the manuscript. AA, AB, and KF revised the manuscript. All authors have read and approved the final manuscript.

## Conflict of Interest

Zimmer GmbH provided the CAD models for the study but was not involved in the study design, collection, analysis, interpretation of data, the writing of this article, or the decision to submit it for publication.

The authors declare that the research was conducted in the absence of any commercial or financial relationships that could be construed as a potential conflict of interest.

## Abbreviations

CAD: computer aided drawing
CT: computed tomography
DF: dual fluoroscopy
MBT: model-based tracking
OA: osteoarthritis
TAR: total ankle replacement.
